# Supplementary figures and images for: Comparison of TENS electrodes and textile electrodes for electrocutaneous warning
Source: PLoS One. 2025 Jun 6;20(6):e0318289. doi: 10.1371/journal.pone.0318289 (PMC12143513; doi:10.1371/journal.pone.0318289)

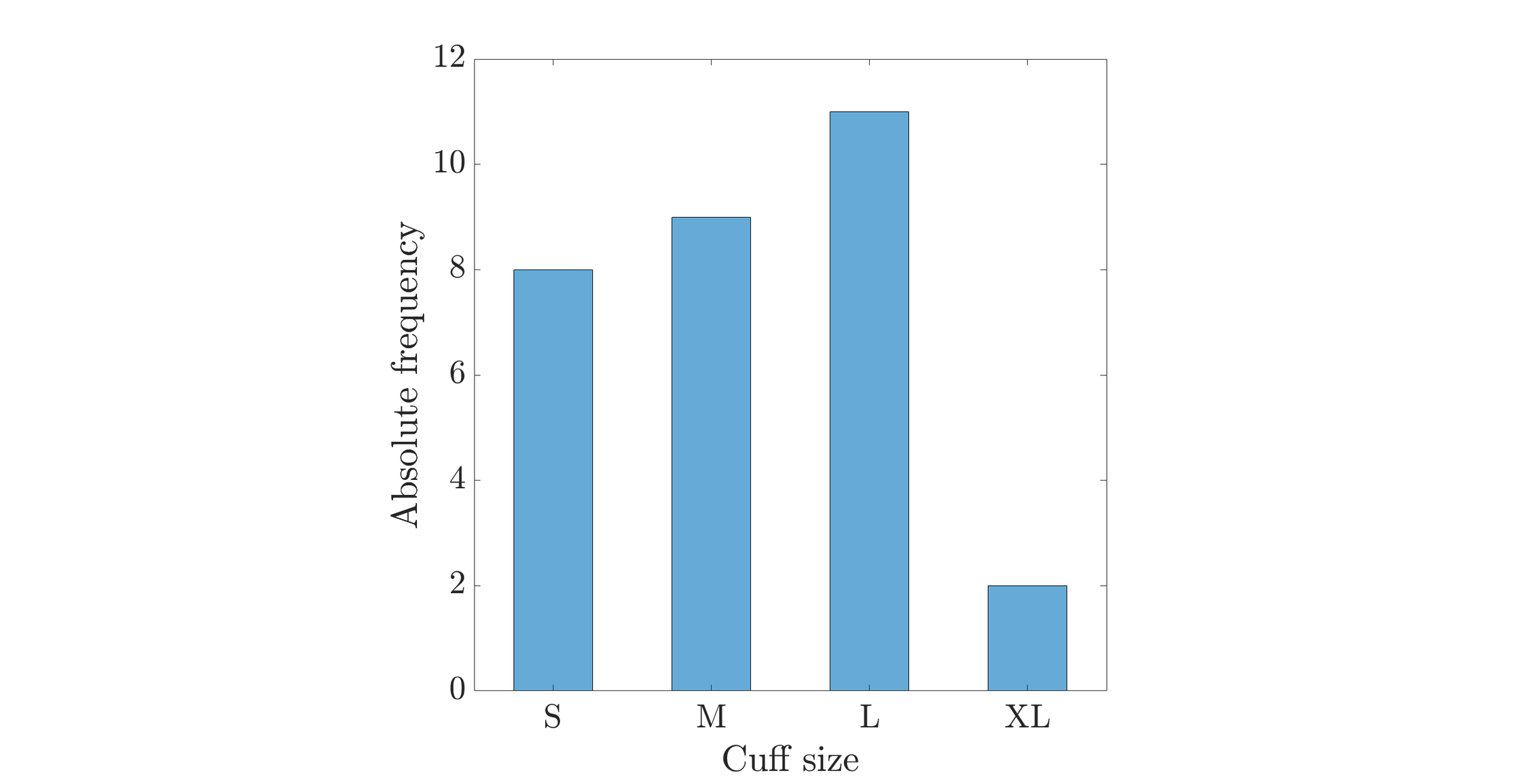

Supplement: S1 Fig — (TIFF) [file pone.0318289.s001.tif]
