# Supplementary material for: Comparison of TENS electrodes and textile electrodes for electrocutaneous warning
Source: PLoS One. 2025 Jun 6;20(6):e0318289. doi: 10.1371/journal.pone.0318289 (PMC12143513; doi:10.1371/journal.pone.0318289)
Supplement: S1 Table — (PDF) [file pone.0318289.s002.pdf]

S1 Table. Number of participants out of 30 without muscle twitches in dependence of the electrode pair and the electrode type.

Electrode type \ Electrode pair no.	**1**	**2**	**3**	**4**	**5**	**6**	**7**	**8**
**TENS**	7	9	6	13	9	5	3	3
**Textile**	17	17	15	11	15	11	10	6
