# Supplementary material for: Comparison of TENS electrodes and textile electrodes for electrocutaneous warning
Source: PLoS One. 2025 Jun 6;20(6):e0318289. doi: 10.1371/journal.pone.0318289 (PMC12143513; doi:10.1371/journal.pone.0318289)
Supplement: S2 Table — (PDF) [file pone.0318289.s003.pdf]

**S2 Table. Number of participants out of 30 with attention and intolerance thresholds $> 25\,\mathbf{mA}$ in dependence of the electrode pair and the electrode type.**

| | | Electrode pair no. | | | | | | | |
|---|---|---|---|---|---|---|---|---|---|
| **Threshold** | **Electrode type** | **1** | **2** | **3** | **4** | **5** | **6** | **7** | **8** |
| **Attention** | **TENS** | 2 | 2 | 2 | 3 | 2 | 2 | 1 | - |
| | **Textile** | 3 | 2 | 2 | 2 | 2 | 2 | 2 | 1 |
| **Intolerance** | **TENS** | 19 | 20 | 20 | 18 | 16 | 17 | 15 | 15 |
| | **Textile** | 14 | 14 | 17 | 16 | 14 | 12 | 15 | 13 |
