# Supplementary material for: Comparison of TENS electrodes and textile electrodes for electrocutaneous warning
Source: PLoS One. 2025 Jun 6;20(6):e0318289. doi: 10.1371/journal.pone.0318289 (PMC12143513; doi:10.1371/journal.pone.0318289)
Supplement: S3 Table — (PDF) [file pone.0318289.s004.pdf]

S3 Table. Number of participants out of 30 and median stimulation amplitudes in mA, where too high transition impedance occurred for textile electrodes in dependence of the threshold and the electrode pair.

	Electrode pair no.							
	**1**	**2**	**3**	**4**	**5**	**6**	**7**	**8**
**Attention threshold**	1	1	-	-	1	2	1	-
**Intolerance threshold**	4	3	-	2	2	6	2	1
**Median stimulation amplitude in mA**	19.95	16.7	-	10.7	15.3	15.65	15.8	22
