# Supplementary material for: Comparison of TENS electrodes and textile electrodes for electrocutaneous warning
Source: PLoS One. 2025 Jun 6;20(6):e0318289. doi: 10.1371/journal.pone.0318289 (PMC12143513; doi:10.1371/journal.pone.0318289)
Supplement: Attachment — Submitted filename: Rebuttal letter_pone_tensvstextile.pdf [file pone.0318289.s005.pdf]

Rebuttal letter

Dear Dr. Agnese Sbrollini and Reviewers,

On behalf of my co-authors and myself, I extend our sincere thanks for your time and effort in reviewing our manuscript titled “Comparison of TENS electrodes and textile electrodes for electrocutaneous warning”. Your detailed comments and constructive feedback have been very helpful in improving our work. We appreciate your thorough evaluations and professional guidance.

Please find our responses to your comments below.

Best regards,

E.-M. Dölker

Dr. Eva-Maria Dölker
Institute for Biomedical Engineering and Informatics
Technische Universität Ilmenau
98693 Ilmenau
eva-maria.doelker@tu-ilmenau.de

Comments to the Academic Editor

1. Please ensure that your manuscript meets PLOS ONE's style requirements, including those for file naming. The PLOS ONE style templates can be found at https://journals.plos.org/plosone/s/file?id=wjVg/PLOSOne_formatting_sample_main_body.pdf and https://journals.plos.org/plosone/s/file?id=ba62/PLOSOne_formatting_sample_title_authors_affiliations.pdf.
Answer: We checked the style requirements and updated the manuscript accordingly.
2. Please note that PLOS ONE has specific guidelines on code sharing for submissions in which author-generated code underpins the findings in the manuscript. In these cases, we expect all author-generated code to be made available without restrictions upon publication of the work. Please review our guidelines at https://journals.plos.org/plosone/s/materials-and-software-sharing#loc-sharing-code and ensure that your code is shared in a way that follows best practice and facilitates reproducibility and reuse.
Answer: Thanks for pointing out. We use only standard Matlab functions for standard statistics and standard visualization purposes which are explained in the manuscript.
3. Thank you for stating the following financial disclosure: [copy in funding statement]. Please state what role the funders took in the study. If the funders had no role, please state: ""The funders had no role in study design, data collection and analysis, decision to publish, or preparation of the manuscript."" If this statement is not correct you must amend it as needed. Please include this amended Role of Funder statement in your cover letter; we will change the online submission form on your behalf.
Answer: We included the amended Funding Statement in our cover letter.
4. When completing the data availability statement of the submission form, you indicated that you will make your data available on acceptance. We strongly recommend all

authors decide on a data sharing plan before acceptance, as the process can be lengthy and hold up publication timelines. Please note that, though access restrictions are acceptable now, your entire data will need to be made freely accessible if your manuscript is accepted for publication. This policy applies to all data except where public deposition would breach compliance with the protocol approved by your research ethics board. If you are unable to adhere to our open data policy, please kindly revise your statement to explain your reasoning and we will seek the editor's input on an exemption. Please be assured that, once you have provided your new statement, the assessment of your exemption will not hold up the peer review process.
Answer: We changed the Data Availability Statement to: All data are in the manuscript and/or supporting information files at https://doi.org/10.5281/zenodo.14833284
5. We note that you have referenced "Dolker EM, Grollich D, Schmauder M, Haueisen J." which has currently not yet been accepted for publication. Please remove this from your References and amend this to state in the body of your manuscript: (Dolker EM, Grollich D, Schmauder M, Haueisen J. [Submitted]") as detailed online in our guide for authors http://journals.plos.org/plosone/s/submission-guidelines#loc-reference-style
Answer: We apologize for the misunderstanding. We now cited the published version on ResearchSquare:<br><br>Dölker EM, Gröllich D, Schmauder M, Haueisen J. Vibration, temperature, and humidity influences on the perception of electrocutaneous stimulation for occupational warning. ResearchSquare [Preprint]. 2024 [cited 2025 Feb 21]: [18 p]. Available from: https://www.researchsquare.com/article/rs-4795941/v1 doi:10.21203/rs.3.rs-4795941/v1
6. We notice that your supplementary tables are included in the manuscript file. Please remove them and upload them with the file type 'Supporting Information'. Please ensure that each Supporting Information file has a legend listed in the manuscript after the references list.
Answer: We removed them accordingly, uploaded them as supporting information and added them to the legend list in the manuscript.
7. We notice that your supplementary figures are uploaded with the file type 'Figure'. Please amend the file type to 'Supporting Information'. Please ensure that each Supporting Information file has a legend listed in the manuscript after the references list.
Answer: We removed them accordingly, uploaded them as supporting information and added them to the legend list in the manuscript.
8. Please review your reference list to ensure that it is complete and correct. If you have cited papers that have been retracted, please include the rationale for doing so in the manuscript text, or remove these references and replace them with relevant current references. Any changes to the reference list should be mentioned in the rebuttal letter that accompanies your revised manuscript. If you need to cite a retracted article, indicate the article's retracted status in the References list and also include a citation and full reference for the retraction notice.
Answer: We reviewed the reference list and made updates according to the comments of the reviewers.

Comments to the Reviewers
Reviewer #1:

1. Kindly add the future scope of the work
2. To enhance the document, it is recommended to incorporate a detailed section on the future scope of the work. This addition should explore potential advancements, applications, or

extensions of the study, emphasizing how it could address existing gaps or open new research avenues. For instance, the future scope could highlight innovative methodologies, interdisciplinary collaborations, or emerging technologies that could further develop the study's findings.

Answer: Thank you for this comment. We reformulated the chapter "Conclusions" as follows:
Our study contributes to the ongoing effort to establish an electrocutaneous stimulation to alert workers in potentially hazardous situations. We successfully demonstrate the application of warning signals through wearable textile electrodes. We compare the novel textile electrode cuff with standard TENS electrodes and demonstrate comparable effectiveness. The textile electrodes showed occasional cases where the electrode-skin transition impedance was too high, which will be addressed through electrode optimization in future work. We found that textile electrodes within the cuff caused less frequent muscle twitches compared to TENS electrodes. To further reduce muscle twitches, future work will optimize the shape and positioning of the electrodes.
We conclude that impedance monitoring is advisable for a future electric warning wearable. In addition, efforts will be made to miniaturize the setup, making it more portable and facilitating the integration of the electrodes into work clothing. Field tests in real work environments will be conducted to assess the system's effectiveness under practical conditions also considering a wider age distribution of participants. Furthermore, long-term applications will be examined, including continuous use over a full 8-hour workday. Beyond occupational safety, the findings of this and future studies may also be relevant for other applications of electrocutaneous stimulation, such as sensory feedback for prosthetics or muscle stimulation.

3.Additionally, it is crucial to update the reference section to include research papers cited from the year 2025. These references should predominantly draw from studies conducted during 2023–2024 to ensure the document reflects the latest advancements and aligns with contemporary scholarly discourse. Integrating these citations will substantiate the relevance of the work and demonstrate its grounding in the most recent research trends.

Answer:
Thank you for your remark. We updated the references list accordingly (changes in red).

The references:
14. Geng B, Yoshida K, Jensen W. Impacts of selected stimulation patterns on the perception threshold in electrocutaneous stimulation. Journal of neuroengineering and rehabilitation. 2011;8:9. doi:10.1186/1743-0003-8-9.

15. Dietrich C, Nehrdich S, Seifert S, Blume KR, Miltner WHR, Hofmann GO, et al. Leg Prosthesis With Somatosensory Feedback Reduces Phantom Limb Pain and Increases Functionality. Frontiers in neurology. 2018;9:270. doi:10.3389/fneur.2018.00270.

16. Chee L, Valle G, Marazzi M, Preatoni G, Haufe FL, Xiloyannis M, et al. Optimally-calibrated non-invasive feedback improves amputees' metabolic consumption, balance and walking confidence. Journal of neural engineering. 2022;19(4). doi:10.1088/1741-2552/ac883b.

17. Pagel A, Arieta AH, Riener R, Vallery H. Effects of sensory augmentation on postural control and gait symmetry of transfemoral amputees: a case description. Medical & biological engineering & computing. 2016;54(10):1579–1589. doi:10.1007/s11517-015-1432-2.

referring to sensory feedback as field of application of electrocutaneous stimulation have been updated by the new references

Cimolato A, Raspopovic S. Closing the sensory feedback loop is necessary for effective neurorehabilitation. PLoS biology. 2024;22(10):e3002866. doi:10.1371/journal.pbio.3002866 .

Malesevic N, Antfolk C. Sensory feedback in upper limb prosthetics: advances and challenges. Nature reviews Neurology. 2024;20(8):449–450. doi:10.1038/s41582-024-00987-3

We also updated

31. Ranatunga KW, Wylie S. Temperature effects on mammalian muscle contraction. Biomedica biochimica acta. 1989;48:S530–5.

by

Malak B, Celichowski J, Drzymala-Celichowska H. The influence of temperature on contractile properties of motor units in rat medial gastrocnemius. Journal of electromyography and kinesiology : official journal of the International Society of Electrophysiological Kinesiology. 2023;68:102738. doi:10.1016/j.jelekin.2022.102738 .

Additionally,

32. Leong GWS, Lauschke J, Rutowski SB, Waite PM. Age, gender, and side differences of cutaneous electrical perceptual threshold testing in an able-bodied population. The journal of spinal cord medicine. 2010;33(3):249–255. doi:10.1080/10790268.2010.11689702.

33. Kemp J, Despr´es O, Pebayle T, Dufour A. Age-related decrease in sensitivity to electrical stimulation is unrelated to skin conductance: an evoked potentials study. Clinical neurophysiology : official journal of the International Federation of Clinical Neurophysiology. 2014;125(3):602–607. doi:10.1016/j.clinph.2013.08.020.

were updated by

Saraiva LHA, Da Viana LS, Pereira LC, Costa RJRM, Holsbach DR. Sex and age differences in sensory threshold for transcutaneous electrical stimulation. Fisioterapia em Movimento. 2022;35. doi:10.1590/fm.2022.35148

Additionally, we added the following references within the introductions section:

Henrique Silva Ribeiro A, Ver¸cosa N, Braga ELC, Micuci AJQR, Germano-Filho PA, Cavalcanti IL. Effect of tetanic stimulation prior to train-of-four monitoring on the time to muscle response stabilization with neuromuscular blockade with rocuronium in patients aged 60 to 80 years: A prospective randomized controlled trial. PloS one. 2024;19(6):e0303161. doi:10.1371/journal.pone.0303161 .

Kim SJ, Worthy A, Lee B, Jafari S, Dyke O, Cho J, et al. Adapting spatiotemporal gait symmetry to functional electrical stimulation during treadmill walking. PloS one. 2024;19(10):e0312285. doi:10.1371/journal.pone.0312285

Lebrun L, Lenoir C, Leone C, van den Broeke EN, Caspani O, Schilder A, et al. Strength, extent and duration of secondary hyperalgesia induced by high-frequency electrical stimulation of the foot compared to the volar forearm of

healthy human volunteers. PloS one. 2025;20(2):e0318934. doi:10.1371/journal.pone.0318934 .

Wang XZ, Wang RK, Liu Q, Shi GX, Mi BH, Liu CZ, et al. Electroacupuncture versus exercise in patients with knee osteoarthritis: Study protocol for a randomized controlled trial. PloS one. 2024;19(6):e0305105. doi:10.1371/journal.pone.0305105 .

Xu S, Huang K, Jiang Q. Evaluation of transcutaneous electrical acupoint stimulation for improving pain and cognitive function in elderly patients around the perioperative period of hip replacement surgery: A meta-analysis. PloS one. 2024;19(10):e0309673. doi:10.1371/journal.pone.0309673 .

Charlton JM, Chang E, Hou SW, Lo E, McClure E, Plater C, et al. Moving in pain - A preliminary study evaluating the immediate effects of experimental knee pain on locomotor biomechanics. PloS one. 2024;19(6):e0302752. doi:10.1371/journal.pone.0302752 .

Jimenez-Garcıa AM, Bonnel G, ´Alvarez-Mota A, Arias N. Current perspectives on neuromodulation in ALS patients: A systematic review and meta-analysis. PloS one. 2024;19(3):e0300671. doi:10.1371/journal.pone.0300671 .

Including a new paragraph in the introduction:
Specialized uses of electrocutaneous stimulation include transcutaneous electrical acupoint stimulation [15, 17, 18], neuromodulation for treatment of patients with amyotrophic lateral sclerosis [20], the investigation of experimental knee pain on locomotor biomechanics [19] or the study of hyperalgesia induced by electrical stimulation [16].

Reviewer #2:

1. The research gap in the abstract was not well defined, in addition to what are the standards and what are their values that the researcher obtained during the work.

Answer: Thank you for this comment. We updated (changes indicated in red) the corresponding section in the Abstract follows:

Electrocutaneous stimulation can be employed to alert workers in potentially hazardous situations. Previous parameter studies used TENS electrodes during the developmental process of the electrical warning system. As a step towards more practicability, we now focus on electrocutaneous stimulation through wearable textile electrodes. In order to determine the feasibility of a novel textile electrode cuff in comparison to previously used TENS electrodes, two studies were conducted.

2. Identify the researcher’s contributions in brief and clear points, in addition to including a paragraph at the end of the introduction that explains the structure of the research in all its sections.

Answer: Thank you for this comment.

We included the researcher’s contributions within the reformulated chapter “Conclusions”:

Our study contributes to the ongoing effort to establish an electrocutaneous stimulation to alert workers in potentially hazardous situations. We successfully demonstrate the

application of warning signals through wearable textile electrodes. We compare the novel textile electrode cuff with standard TENS electrodes and demonstrate comparable effectiveness. The textile electrodes showed occasional cases where the electrode-skin transition impedance was too high, which will be addressed through electrode optimization in future work. We found that textile electrodes within the cuff caused less frequent muscle twitches compared to TENS electrodes. To further reduce muscle twitches, future work will optimize the shape and positioning of the electrodes.
We conclude that impedance monitoring is advisable for a future electric warning wearable. In addition, efforts will be made to miniaturize the setup, making it more portable and facilitating the integration of the electrodes into work clothing. Field tests in real work environments will be conducted to assess the system's effectiveness under practical conditions also considering a wider age distribution of participants. Furthermore, long-term applications will be examined, including continuous use over a full 8-hour workday.
Beyond occupational safety, the findings of this and future studies may also be relevant for other applications of electrocutaneous stimulation, such as sensory feedback for prosthetics or muscle stimulation.

We updated (changes indicated in red) the corresponding paragraph in chapter "Introduction" as follows:

The aim of the current study is to compare a novel set of 8 textile electrode pairs mounted within a novel cuff with the previously used TENS electrodes. For that purpose, two studies were conducted. In the first study (n = 30), the perception thresholds, qualitative and spatial perceptions were compared between textile and TENS electrodes. In the second study (n = 36), the textile electrodes were investigated during vibration and under the presentation of a warning signal to investigate whether the results were comparable to the ones obtained with the TENS electrodes.
The paper is organized as follows. In section Materials and methods, we present the study groups of the two studies, the experimental setup for electrocutaneous stimulation, properties of TENS and textile electrodes, definition and determination of thresholds, qualitative and spatial perceptions, and the application of vibration. In this section, also the experimental paradigms of both studies are described. The section is finished with a description of the statistics. The section Results shows perception, attention, muscle twitch, and intolerance thresholds as well as qualitative and spatial perceptions for TENS and textile electrodes of study 1 and the same thresholds for the textile electrodes during rest and under vibration of study 2. Additionally, the results of the application of a warning signal during mechanical vibration are presented. Results are discussed in the Discussion section. In the last section, we give the conclusions and directions for future work.

3. Clarify future work in the conclusions, in addition to relying on numerical values for the results in building clear conclusions for the reader.

Answer: Thank you for this comment. We reformulated the chapter "Conclusions" as follows:
Our study contributes to the ongoing effort to establish an electrocutaneous stimulation to alert workers in potentially hazardous situations. We successfully demonstrate the application of warning signals through wearable textile electrodes. We compare the novel textile electrode cuff with standard TENS electrodes and demonstrate comparable effectiveness. The textile electrodes showed occasional cases where the electrode-skin transition impedance was too high, which will be addressed through electrode optimization in future work. We found that textile electrodes within the cuff caused less frequent muscle twitches compared to TENS electrodes. To further reduce muscle twitches, future work will optimize the shape and positioning of the electrodes.
We conclude that impedance monitoring is advisable for a future electric warning wearable. In addition, efforts will be made to miniaturize the setup, making it more portable and facilitating the integration of the electrodes into work clothing. Field tests in real work

environments will be conducted to assess the system’s effectiveness under practical conditions also considering a wider age distribution of participants. Furthermore, long-term applications will be examined, including continuous use over a full 8-hour workday. Beyond occupational safety, the findings of this and future studies may also be relevant for other applications of electrocutaneous stimulation, such as sensory feedback for prosthetics or muscle stimulation.

4. Arrange references in one format

Answer: Thank you for your comment. We arranged the references in one format.
